# Frequency of Hereditary Hemochromatosis Gene *(HFE)* Variants in Sri Lankan Transfusion-Dependent Beta-Thalassemia Patients and Their Association With the Serum Ferritin Level

**DOI:** 10.3389/fped.2022.890989

**Published:** 2022-07-12

**Authors:** Padmapani Padeniya, Hemali Goonasekara, Gayan Abeysekera, Rohan Jayasekara, Vajira Dissanayake

**Affiliations:** ^1^Department of Anatomy, Faculty of Medicine, University of Kelaniya, Ragama, Sri Lanka; ^2^Department of Anatomy, Faculty of Medicine, University of Colombo, Colombo, Sri Lanka

**Keywords:** transfusion dependent thalassemia, c.845G>A, c.187C>G, ferritin, ARMS-PCR, hereditary hemochromatosis

## Abstract

**Introduction:**

Co-inheritance of hereditary hemochromatosis (*HFE*) gene variants *p*. C282Y and *p*.H63D worsen iron overload in transfusion-dependent thalassemia. Data on the *HFE* gene variants in Sri Lankan patients with thalassemia have not been extensively studied. This study aimed to analyze the *p*.C282Y and *p*.H63D variants in transfusion-dependent beta (β) and HbE/β-thalassemia patients and establish an association between these variants and their serum ferritin levels.

**Materials and Methods:**

A total of 125 transfusion-dependent β-thalassemia major and HbE/β thalassemia patients were tested for the c.845G>A (*p*.C282Y) and c.187C>G (*p*.H63D) *HFE* gene variants using the multiplex Amplification Refractory Mutation System Polymerase Chain Reaction method. For phenotype-genotype correlation, serum ferritin levels, the erythrocyte sedimentation rate (ESR), and C-reactive protein (CRP) levels were measured. The standard descriptive statistics were used for data analysis.

**Results:**

The study cohort consisted of transfusion-dependent 123 β-thalassemia and 2 HbE/β-thalassemia patients. The *p*.C282Y variant was not detected in any patient; allele frequency for the wild type (c.845GG) was 100%. Twenty-three patients were heterozygous for the *p*.H63D variant allele, and the allele frequencies were c.187CC 91.8%, c.187CG 9.2%, and c.187GG 0%. The mean serum ferritin level was relatively higher (mean level 4,987 ng/ml) in the *p*.H63D heterozygous (c.187CG) group compared to the wild type (c.187CC) group (mean level 4,571 ng/ml), but the difference was statistically not significant (*p* = 0.865). Among the total study population, CRP, ESR, and serum glutamine aspartate transaminase (SGPT) were elevated in 9 (7.2%), 65 (52%), and 82 (65.6%) patients, respectively. Among the *p*.H63D c.187CG group, elevated CRP, ESR, and SGPT were present in 5 (5%), 15 (12%), and 18 (14.4%) patients, respectively. The detected sample number was low to correlate with the confounding effect of inflammatory disorders and liver damage on the serum ferritin levels.

**Conclusions:**

The *HFE* gene variant *p*.C282Y is unlikely to cause iron overload in the Asian β-thalassemia patients; the rarity of this variant in the study cohort replicates the findings of other South Asian population studies of this variant. The presence of the *p*.H63D variant could be a potential risk factor for iron overload in the β-thalassemia patients. A more extensive cohort study is required to validate this finding.

## Introduction

The phenotypic diversity of beta (β)-thalassemia is associated with genetic modifiers and environmental factors. Primary genetic modifiers are the wide range of β-globin mutations principally affecting β-globin chain synthesis. Secondary modifiers also affect β-globin synthesis through variations in the alpha (α)-globin or gamma (γ*)-*globin synthesis. Tertiary modifiers are not associated with β-globin synthesis but alter the complications of the disease. Tertiary modifiers include distinct genetic polymorphisms co-selected with β-thalassemia, further adjusting the phenotype by modifying the complications. Some of these recognized complications are iron overload, hyperbilirubinemia, and osteoporosis (1–3).

In patients with transfusion-dependent thalassemia, iron overload following 2–3 years of initiation of blood transfusions is inevitable. Each 500 ml of packed red cell contains 250 mg of elemental iron, and repeated transfusions will saturate the available transferrin level in the circulation, favoring non-transferrin bound iron (NTBI), a toxic compound to be formed. This NTBI generates highly reactive hydroxyl radicals resulting in oxidative damage to various cellular components, such as lipids, proteins, and nucleic acids, causing tissue destruction (4–7). Hepcidin, a small peptide hormone synthesized by the liver, is the primary regulator of iron movement into plasma. When the hepcidin level is very low or absent in the plasma, as in iron deficiency anemia, iron is diverted to plasma from enterocytes and macrophages through the ferroportin transport mechanism (4, 8). Since β-thalassemia major patients have low hepcidin levels in their circulation, it results in an increased level of plasma free iron; iron overload, triggering tissue damage (4).

The coexistence of *HFE* gene-associated hereditary hemochromatosis and β-thalassemia can exacerbate iron overload and iron-related complications in patients with β-thalassemia. It is observed that hemochromatosis is frequently associated with β-thalassemia. Several studies have revealed that the interaction of hereditary hemochromatosis with β-thalassemia can have an exaggerated response in iron absorption and storage in these patients (9–16). In patients with *HFE*-associated hereditary hemochromatosis, two common missense mutations; *c*.845G>A (*p*.C282Y; rs1800562) and *c*.187C>G (*p*.H63D; rs1799945), have been described. There is a significant ethnic variation observed in the distribution of these variants; the *p*.C282Y mutation is mainly limited to the North European region. The prevalence of this variant is considered very low in Australian, African, and Asian populations. The *p*.H63D mutation is shown to have a cosmopolitan distribution across the world with a frequency of 3.3%−15.2% (17–19).

The degree of iron overload can be evaluated either by assessing serum ferritin levels or liver iron concentration (LIC). Assessing LIC using liver biopsy is the gold standard method and the most reliable body iron assessment indicator. Yet its invasive nature with potential morbidity and mortality (<1 in 10,000 cases), poor patient compliance, and sampling error have hindered its routine clinical use (14, 20–23). Serum ferritin levels generally represent body iron stores and have shown to be a convenient and reliable method to assess body iron stores. Furthermore, serial measurements help determine trends of the iron overload (24). However, serum ferritin is an acute-phase reactant and is elevated non-specifically in acute or chronic inflammatory states (25) and, therefore, is an unreliable predictor of body iron stores in the presence of inflammation. Other markers of inflammation, such as C-reactive protein (CRP), would help to eliminate this confounding factor when assessing body iron stores (26, 27). Present-day magnetic resonance imaging (MRI)-based techniques are the most extensively used techniques for LIC estimation. Universal unavailability and economic constraints have resulted in the underutilization of MRI-based assessment of body iron stores in routine clinical practice (28, 29). Serum glutamine aspartate transaminase (SGPT) is a hepatocyte-specific enzyme. It is released into the bloodstream following hepatocyte injury and, therefore, is routinely used as a marker of liver disease (30).

The objective of this study was to genotype *p*.C282Y and *p*.H63D variants of the *HFE* gene in transfusion-dependent β-thalassemia and HbE/β-thalassemia patients and correlates the mutation status with their serum ferritin levels.

## Materials and Methods

### Study Population

This study was a prospective study. Patient recruitment was done from two sites. A total of 125 patients with β-thalassemia and HbE/β-thalassemia who were transfusion-dependent from the Lady Ridgway Hospital, Colombo (a tertiary care children's hospital) and the Thalassemia center in the Teaching Hospital, Anuradhapura were selected for the study. Ethical approval to conduct the study was obtained from the Ethics Review Committee (ERC) of the Faculty of Medicine, the University of Colombo, Sri Lanka (Ref. No: EC-11-127), and the Lady Ridgway Hospital Ethics review Committee, Colombo, Sri Lanka. This study was conducted in accordance with the declaration of Helsinki. Patients and their parents/guardians were interviewed to gather demographic and clinical data following informed written consent. At the time of recruitment, a 10 ml volume venous blood sample was obtained from each study participant for *HFE* gene genotyping and biochemical analysis of serum ferritin, CRP, erythrocyte sedimentation (ESR), and SGPT levels.

### Biochemical Testing

In a commercial laboratory, serum ferritin was measured by a solid-phase, two-site chemiluminescent enzyme immunometric assay. This test had been validated as per the World Health Organization's second international standard for ferritin. The CRP, ESR, and SGPT levels were done to exclude coexisting inflammatory conditions and liver disease, respectively. All the investigations were done in the same laboratory to minimize inter-laboratory variations. According to the published laboratory standards, reference ranges for pediatric and adolescent populations were considered.

### Molecular Genetic Testing

Promega Wizard^®^ Genomic DNA purification kit was used for DNA extraction; and the protocol was carried out according to the manufacturer's advice. A multiplex Amplification Refractory Mutation System Polymerase Chain Reaction (ARMS-PCR) method was used to detect the *HFE* gene *p*.C282Y mutation as described previously (31). For the *HFE* gene *p*.H63D mutation, primers were designed at the genetic laboratory of the Human Genetics Unit, Faculty of Medicine, University of Colombo.

The primers used to genotype the *p*.H63D mutation were as follows.

63Fw-AGCTGT TCGTGTTCTATGATC;
63F4-AGCTGTTCGTGTTCTATGATG;
63R3-CTGTGGTTGTGATTTTCCATAA.

### Statistical Analysis

The distribution of continuous variables was expressed as mean (SD), and categorical variables were presented as frequencies. The *p*-value <0.05 was considered to be statistically significant. The independent Student *t*-test was considered for testing the statistical differences between the two groups. All descriptive and analytical statistics were calculated with R programming language version 3.4.2.

## Results

Of the total study population of 125, 60 (48%) patients were male; the male to female ratio was 60:65. The study cohort consisted of transfusion-dependent 123 β-thalassemia patients and 2 HbE/β-thalassemia patients. The mean age of the study cohort was 8.86 years (SD ± 4.7), and the age range varied between 9 months to 23.5 years. Thirty-four (27%) patients in the study cohort were over 18 years.

Analysis of a three-generation pedigree revealed that 33 (26.4%) patients had third-degree parental consanguinity. However, the majority (67.2) did not have a history of consanguinity. The family history of consanguinity was not recorded in eight (6.4%) patients. The molecular diagnosis was available in 94 (75%) patients. *HBB* genotype distribution and their frequencies are shown in Table 1. The molecular diagnosis was not available in 31 (25%) patients (Table 1).

**Table 1:** The *HBB* genotype distribution and their frequencies.

***HBB*** **genotype**	***N*** **(%)**
**Homozygosity**	
c.92+5G>C	69 (55.2%)
c.92+1G>A	7 (5.6%)
c.126_129delCTTT	1 (0.8%)
c.51delC	1(0.8%)
c.27_28insG	1(0.8%)
**Compound heterozygosity**	
c.92+5G>C; c.126_129delCTTT	5 (4%)
c.92+5G>C; c.92+1G>A	5 (4%)
c.92+5G>C; g.71609_72227del619	1 (0.8%)
c.92+5G>C; c.79G>A	2 (1.6%)
c.92+5G>C; c.46delT	2 (1.6%)

In most patients (58%), blood transfusion was initiated at 6 months. The range of time taken to initiation of the transfusion regimen was between 2 months and 2 years. The mean pre-transfusion hemoglobin level of this study cohort was 8.82 g/dl (SD ± 0.85), and a majority received their monthly blood transfusion when the pre-hemoglobin level was 8.7 g/dl. Of the 125 patients, 17 (13.6%) had undergone splenectomy at their early ages, and cholecystectomy had been performed in one (0.8%) patient.

The mean serum ferritin level was 4,628.5 ng/ml (SD ± 2,614), and the ferritin level ranged from 157 to 12,470 ng/ml. Serum ferritin reports of four patients were not available for analysis. Complications related to iron overload were common in the study group; seven (5.6%) patients had diabetes mellitus at recruitment. Hypothyroidism was reported in five (4%) patients, and one (0.8%) patient had hypoparathyroidism. Two (1.6%) patients in the study cohort had both diabetes mellitus and hypothyroidism. The CRP level was high in nine (7.2%) participants; the mean CRP was 2.2 mg/L. The ESR was elevated in 65 (52%) patients; the mean ESR was 21.7 mm/1st h. The SGPT was elevated in 82 (65.6%), and the mean SGPT was 91 U/L.

**Figure 1:**
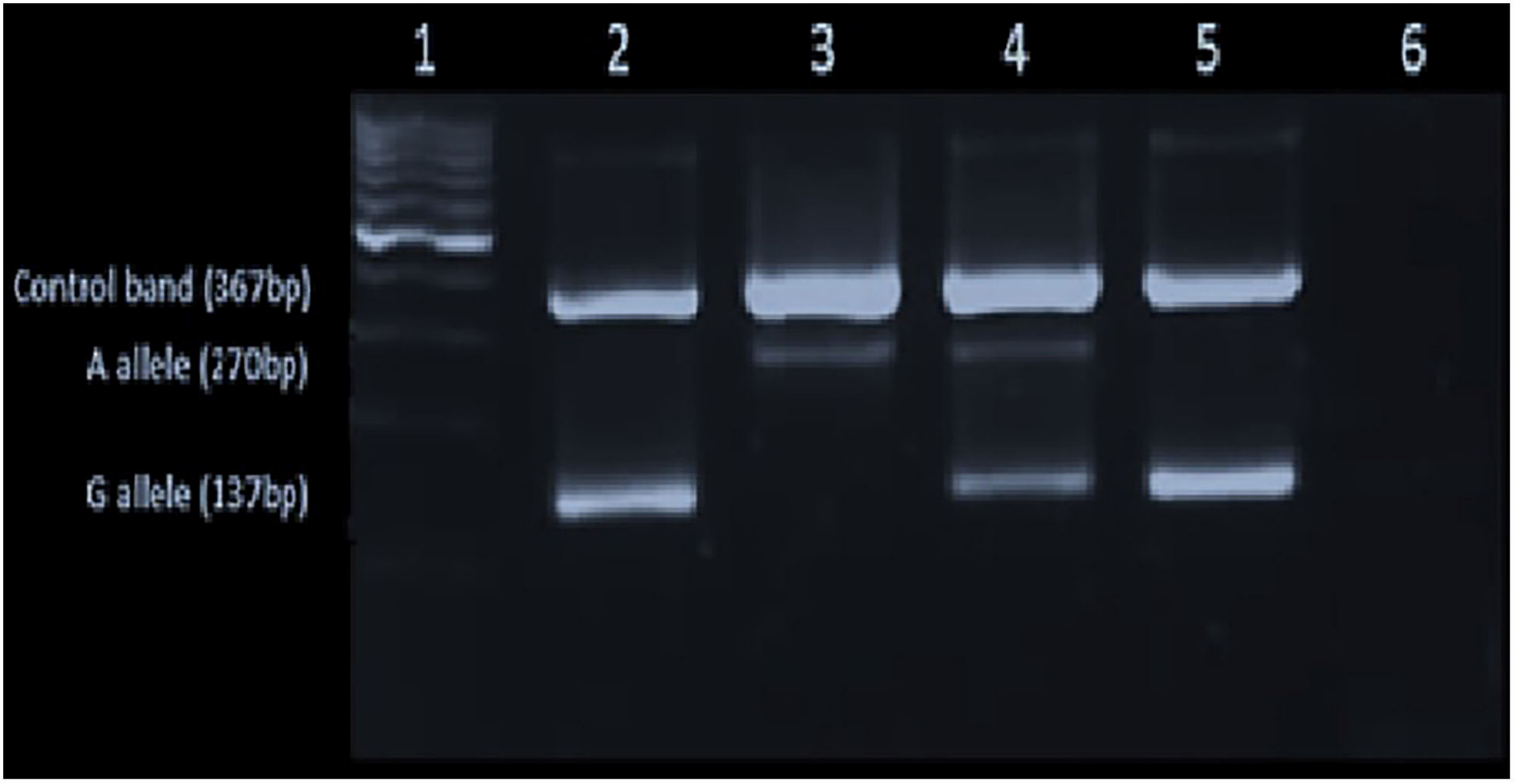
Gel image showing the p.C282Y [c.845G>A] mutation: Lane1-L6 – 100 bp ladder in the 1st lane, homozygote for wild type (GG) in the 2nd lane, the homozygote for mutant A allele in (AA) the 3rd lane, heterozygous for the mutant allele (GA) in the 4th lane and the negative control (GG) and the blank in the 5th and 6th lanes, respectively.

**Figure 2:**
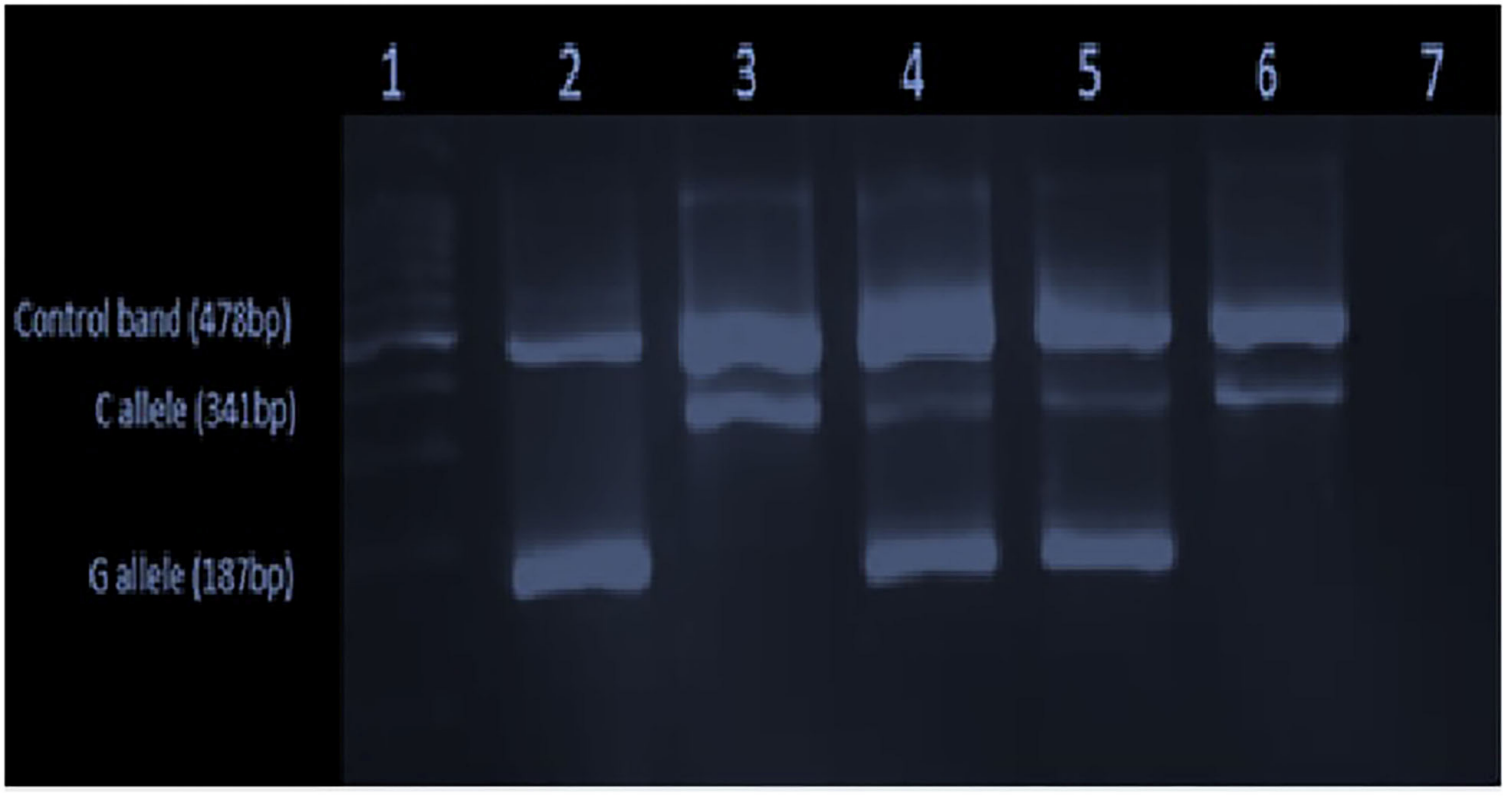
Gel image showing the p.H63D [c.187C>G] mutation: 100 bp ladder in the 1st lane, homozygote for mutant G allele (GG) in the 2nd lane, the homozygote for wild type (CC) in the 3rd lane, heterozygous for the mutant allele (CG) in the 4th lane and the positive control (CG) in the 5th and the negative control and the blank in the 6th and the 7th lanes, respectively.

None of the study participants carried the *p*.C282Y variant allele. All study participants (100%) were the wild-type variant (c.845GG). The *p*.H63D variant allele was detected in the heterozygous state [c. 187CG] in 23 (18.4%) patients and allele frequencies were c.187CC – 91.8%, c.187CG – 9.2%, and c.187GG – 0%. The genotype distribution was in accordance with Hardy–Weinberg equilibrium (32). The agarose gel image results are shown in Figures 1, 2.

**Figure 3:**
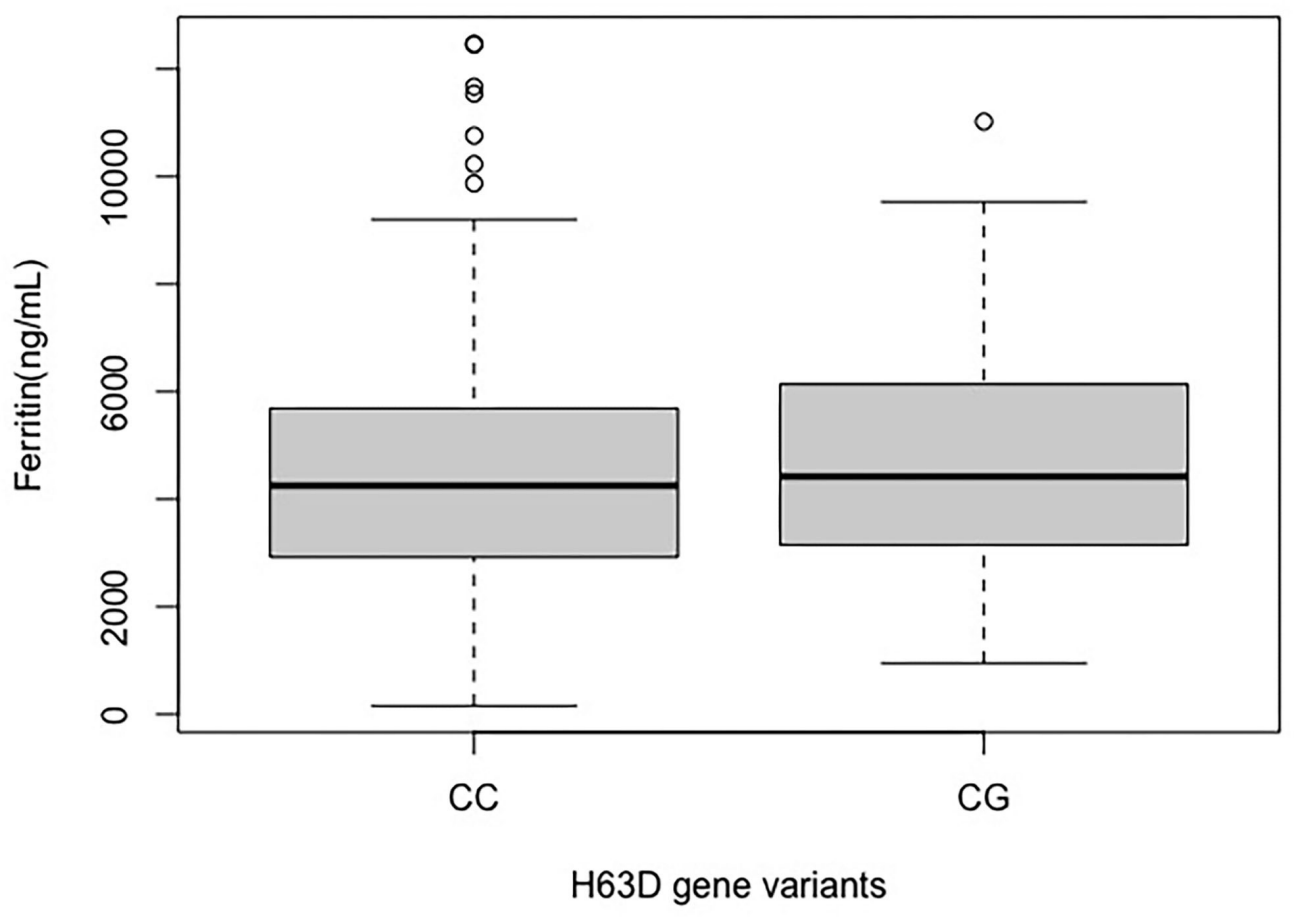
Distribution of serum mean ferritin in H63D wild-type allele (CC) and H63D variant allele (CG) groups.

The mean serum ferritin levels were compared between the patients with the wild-type allele for *p*.H63D [c. 187CC] and the variant allele for *p*.H63D [c. 187CG]. Although the mean serum ferritin levels were high in both groups, the difference was not statistically significant (*p*-value = 0.865; Figure 3).

The comparison of the mean serum ferritin levels with the genotyping data is depicted in Table 2.

**Table 2:** Mean serum ferritin levels and genotype of the *p*.H63D mutation.

**Genotype**	**Number of patients**	**Mean ferritin level (ng/ml)**	**SEM**
CC	98	4,571	265
CG	23	4,987	541

*CC-homozygous for the wild type, CG-heterozygous for the mutant allele*.

Of the nine (7.2%) patients with elevated CRP, four (3.2%) patients carried the wild-type variant (c.187CC) of the *p*.H63D mutation, while five (5%) participants carried the mutant variant (c.187CG). Of the 65 (52%) patients with high ESR, 50 (40%) participants carried the wild-type variant of the *p*.H63D mutation, and 15 (12%) had the mutant allele. Eighty-two (65.6%) patients who had high SGPT, 64 (51.2%) had the wild-type variant, and 18 (14.4%) had the mutant variant of the *p*.H63D mutation.

## Discussion

This study aimed to determine the allele frequency of the common variants *p*.C282Y and *p*.H63D in the *HFE* gene in a cohort of transfusion-dependent thalassemia patients and to determine the genotype–phenotype correlation between the variant status and body iron stores.

None of the patients in our study cohort harbored the *p*.C282Y (*c*.845G>A) variant; hence the allele frequency was 0%. This finding was similar to other populations in Asia (17, 33). Regarding the *p*.H63D (*c*.187C>G) variant, of the total study cohort, 23 patients were heterozygous for the variant allele (CG); hence the allele frequency was 9.2%. The prevalence of the *c*.187C>G variant had previously been analyzed in Sri Lankan cohorts. Following analysis of 109 chromosomes (218 alleles), Rochette et al. (33) reported the allele frequencies of *p*.C282Y and *p*.H63D variants as 0.8 and 10.8%, respectively. Rochette and his colleagues further reported a single case, a compound heterozygote for both mutations. After evaluating 130 referrals sent from Sri Lanka for hemoglobinopathy diagnosis, Merryweather-Clarke concluded that the *p*.C282Y mutation was absent on the island, and the frequency of the *p*.H63D mutation was 9.2% (34) (Table 3).

**Table 3:** Comparison of the allele frequencies of the mutant alleles in the *HFE* gene with the current study.

**Author of the study**	**Number of alleles studied**	***p*** **.C282Y**	***p*** **.H63D**
Merryweather-Clarke et al. (34)	260	0%	9.2%
Rochette et al. (33)	218	0.08%	10.8%
Current study	250	0%	9.2%

### Correlation Between Serum Ferritin and the *p*.H63D Genotype

A relative, but not a statistically significant, difference between the mean serum ferritin levels was present in *p*.H63D heterozygotes (c.187CG). Melis et al. (11) and their colleagues had investigated the correlation between the *p*.H63D mutation and the serum ferritin levels previously. As per their study, serum ferritin level was higher in homozygous patients for the *p*.H63D variant than in patients with the heterozygous variant. The study had concluded that the *p*.H63D mutation has a modifying outcome on iron absorption. Similar to our study findings Melis and his colleagues could not determine a significant difference in the mean serum ferritin levels between the heterozygous variant allele group (GC) vs. the wild-type allele group.

Piperno et al. (13) have concluded that the coinheritance of β-thalassemia minor along with *c*.845G>A homozygous (AA) status exaggerated the clinical picture and is more likely to develop severe iron-related complications. However, the study could not find a significant correlation between the presence of heterozygous status for *c*.845G>A and *c*.187C>G variant alleles and their serum ferritin levels (13). After studying a 168 Brazilian β-thalassemia heterozygous cohort, Oliveira et al. (10) concluded that the clinical picture is worsened when the *c*.845G>A variant is co-inherited in β-thalassemia carriers.

A summary of studies assessing *HFE* gene variants and body iron status in patients with β-thalassemia major and intermedia is given in Table 4.

**Table 4:** The *HFE* gene mutation analysis: summary of previous studies done on β thalassemia major and intermedia patients and the present study.

**Number**	**Study design/authors**	**Type of thalassemia**	**Country**	***N***	***p*** **.C282Y**	***p*** **.H63D**	**Correlation with serum ferritin**
1	Case/control Longo et al. (12)	Major	Italy	71	1.4%	12.7%	No
2	Case/control Kaur et al. (35)	Major	India	75	4%	12.6%	No
3	Case/control Enein et al. (15)	Major	Egypt	50	0%	10%	Yes
4	Cases only Hashmi et al. (16)	Major	Pakistan	274	N/A	10%	N/A
5	Cases only Rees et al. (9)	Intermedia	Mix ethnic group	81	0.6%	0%	N/A
6	Cases only Cappellini et al. (14)	Intermedia	Italy	37	_	0%	N/A
7	Descriptive Current study	Major	Sri Lanka	125	0%	9.2%	No

*N, number of patients; N/A, not available*.

Except for one study (15), irrespective of the patient cohort, i.e., whether they were transfusion-dependent thalassemia or thalassemia intermedia, none of the other studies demonstrated a significant correlation between the serum ferritin levels and the heterozygous state for the *p*.H63D variant, as was found in our study. The study done by Enein et al. (15) in an Egyptian thalassemia cohort revealed significantly higher serum ferritin and serum iron levels in transfusion-dependent thalassemia patients in the presence of the *p*.H63D variant allele in heterozygous state.

The main limitation of this study was that the detected *HFE* gene variant allele was limited to heterozygous *p*.H63D variant allele, and the number of positives being only 23 (18.4%). The statistically non-significant mean serum ferritin level between the groups with and without the variant allele could be due to the low sample number.

It is well recognized that hyperferritinemia occurs due to factors extraneous to iron overloading factors associated with thalassemia. Thus, supportive biochemical workup can help identify and eliminate the confounding factors (27). Biochemical evaluations of CRP, SGPT, and ESR were performed on the study participants to assess the commonly occurring confounding factors such as infections, liver parenchymal damage, and inflammatory disorders, respectively. Since the cohort giving elevated values was small in number in the variant positive subgroup, the sample size in each subgroup, the confounding effect of inflammatory disorders and liver damage on the serum ferritin level were unable to be assessed precisely.

In conclusion, the variant *p*.C282Y is unlikely to cause iron overload in Asian β-thalassemia patients; the rarity of this variant in the study cohort replicates the findings of other South Asian population studies of this variant. The presence of the *p*.H63D variant could be a potential risk factor for iron overload in β-thalassemia patients. A more extensive cohort study is required to validate this finding and determine its usefulness as a routine test to predict the risk of iron overloading in β-thalassemia patients.

## Data Availability Statement

The original contributions presented in the study are included in the article/supplementary material, further inquiries can be directed to the corresponding author.

## Ethics Statement

The studies involving human participants were reviewed and approved by Ethics Review Committee (ERC) of the Faculty of Medicine, University of Colombo, Sri Lanka. Written informed consent to participate in this study was provided by the participants' legal guardian/next of kin.

## Author Contributions

All authors listed have made a substantial, direct, and intellectual contribution to the work and approved it for publication.

## Funding

This study was supported by the NOMA scholarship to follow the MSc in Clinical Genetics funded by the Norad and managed by the Centre for International co-operation in Higher Education, Oslo, Norway.

## Conflict of Interest

The authors declare that the research was conducted in the absence of any commercial or financial relationships that could be construed as a potential conflict of interest.

## Publisher's Note

All claims expressed in this article are solely those of the authors and do not necessarily represent those of their affiliated organizations, or those of the publisher, the editors and the reviewers. Any product that may be evaluated in this article, or claim that may be made by its manufacturer, is not guaranteed or endorsed by the publisher.
